# Comparison of Clinical Efficacy of Lateral and Lateral and Medial Double-plating Fixation of Distal Femoral Fractures

**DOI:** 10.1038/s41598-018-23268-8

**Published:** 2018-03-20

**Authors:** Zhibiao Bai, Shichang Gao, Zhenming Hu, Anlin Liang

**Affiliations:** Department of Orthopaedics, The first Affiliated Hospital of Chongqing Medical University, Chongqing, 400016 China

## Abstract

The present study was performed to compare the clinical efficacy of lateral plate and lateral and medial double-plating fixation of distal femoral fractures and explore the indication of lateral and medial double-plating fixation of the distal femoral fractures. From March 2006 to April 2014, 48 and 12 cases of distal femoral fractures were treated with lateral plate (single plate) and lateral and medial plates (double plates), respectively. During the surgery, after setting the lateral plate for the distal femoral fractures, if the varus stress test of the knee was positive and the lateral collateral ligament rupture was excluded, lateral and medial double-plating fixation was used for the stability of the fragments. All the patients were followed up at an average period of 15.9 months. The average operation time, the intraoperative hemorrhage and the fracture union time of the two groups were compared. One year after operation, knee function was evaluated by the Kolmert’s standard. There was no significant difference in the average operation time, intraoperative hemorrhage, fracture healing time and excellent and good rates of postoperative knee function between two groups. Positive Varus stress test during operation can be an indication for lateral and medial double-plating fixation of distal femoral fractures.

## Introduction

Clinically, fractures within 9 cm of the articular surface of distal femoral are defined as distal femoral fractures^1^, which account for 6% of all femoral fractures and men under 40 years old and women over 50 years old are more susceptible to^2^. Distal femoral fractures are mostly caused by high-energy injury, such as falling injury and traffic accidents, and fractures are often severely comminuted, which may be associated with injuries to knee joint structural and other parts, such as tibial plateau fractures, traumatic brain injury and pelvic fractures. At present, the distal femoral fractures are mainly fixed by the lateral anatomical locking plate, but lateral and medial double-plating fixation is also suggested by some researchers^3–5^.

However, there is no conclusion on the indication of lateral and medial double-plating fixation for treating distal femoral fractures. To explore the indications for treatment of distal femoral fractures with lateral and medial double-plating fixation and its clinical efficacy, we followed up a total of 58 patients, with 60 cases of distal femoral fractures fixed in our hospital from March 2006 to April 2014, of which 48 cases were fixed by lateral plate and 12 were fixed by the lateral and medial double-plating fixation.

## Results

**Figure 1:**
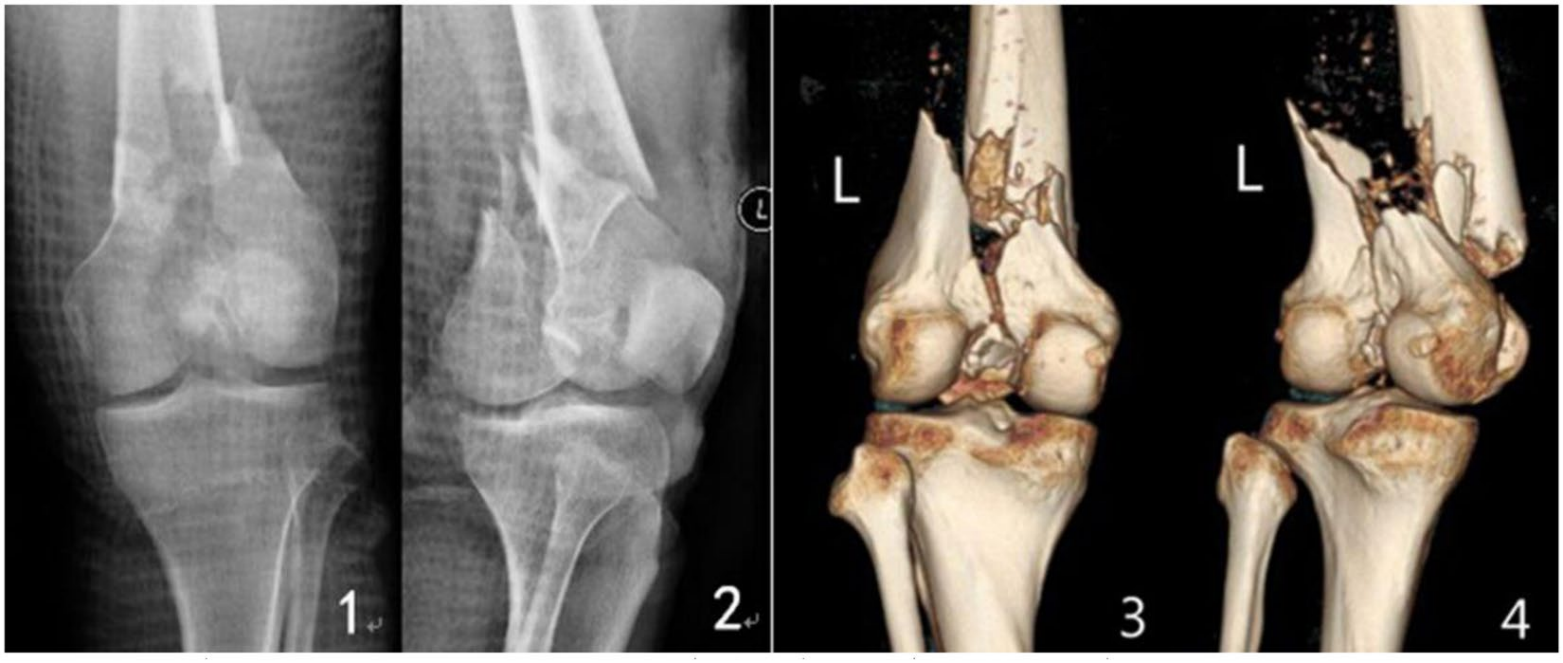
1, 2, 3, 4 the preoperative X-ray and CT three-dimensional reconstruction of femur.

**Figure 2:**
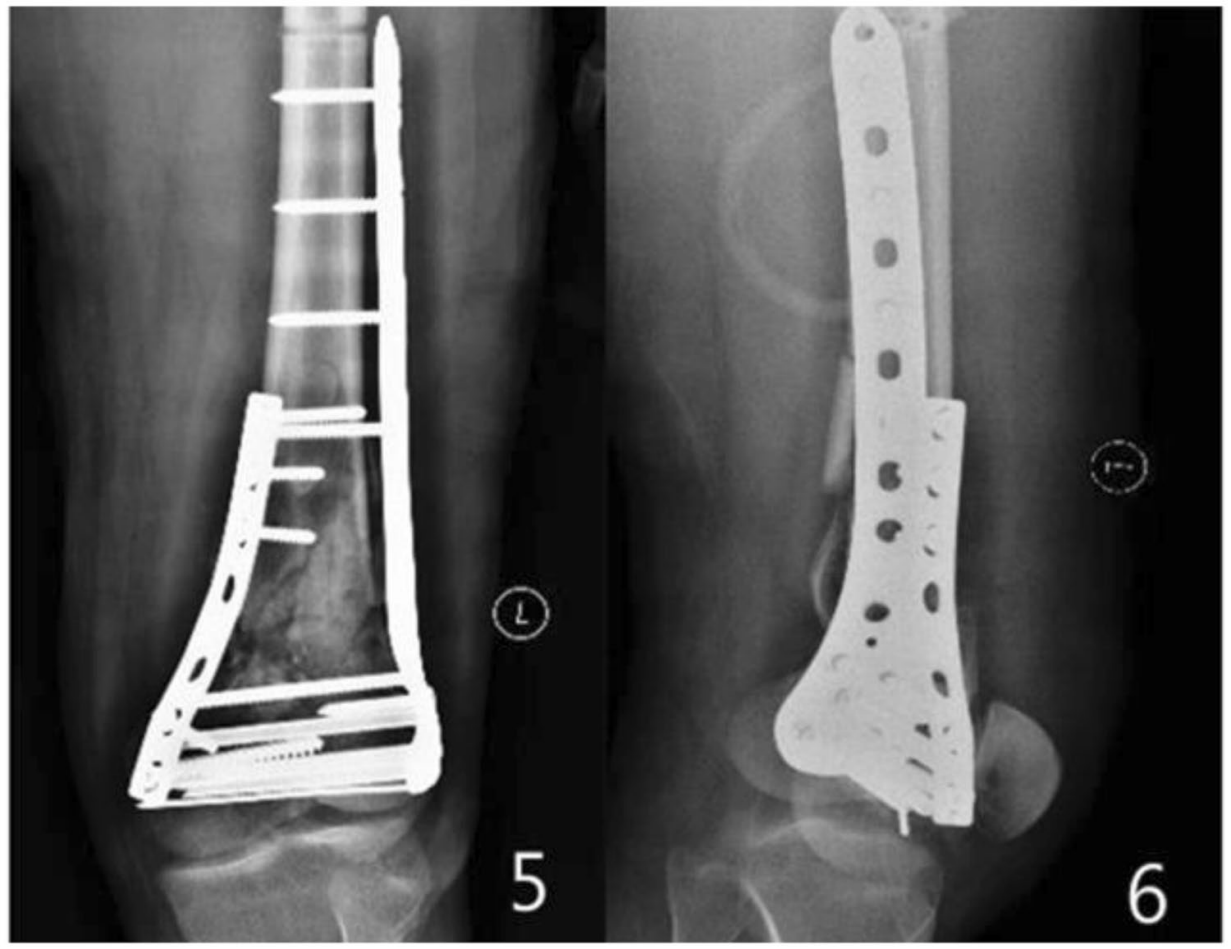
5, 6 the X-ray of the femur, taken two days after operation.

**Figure 3:**
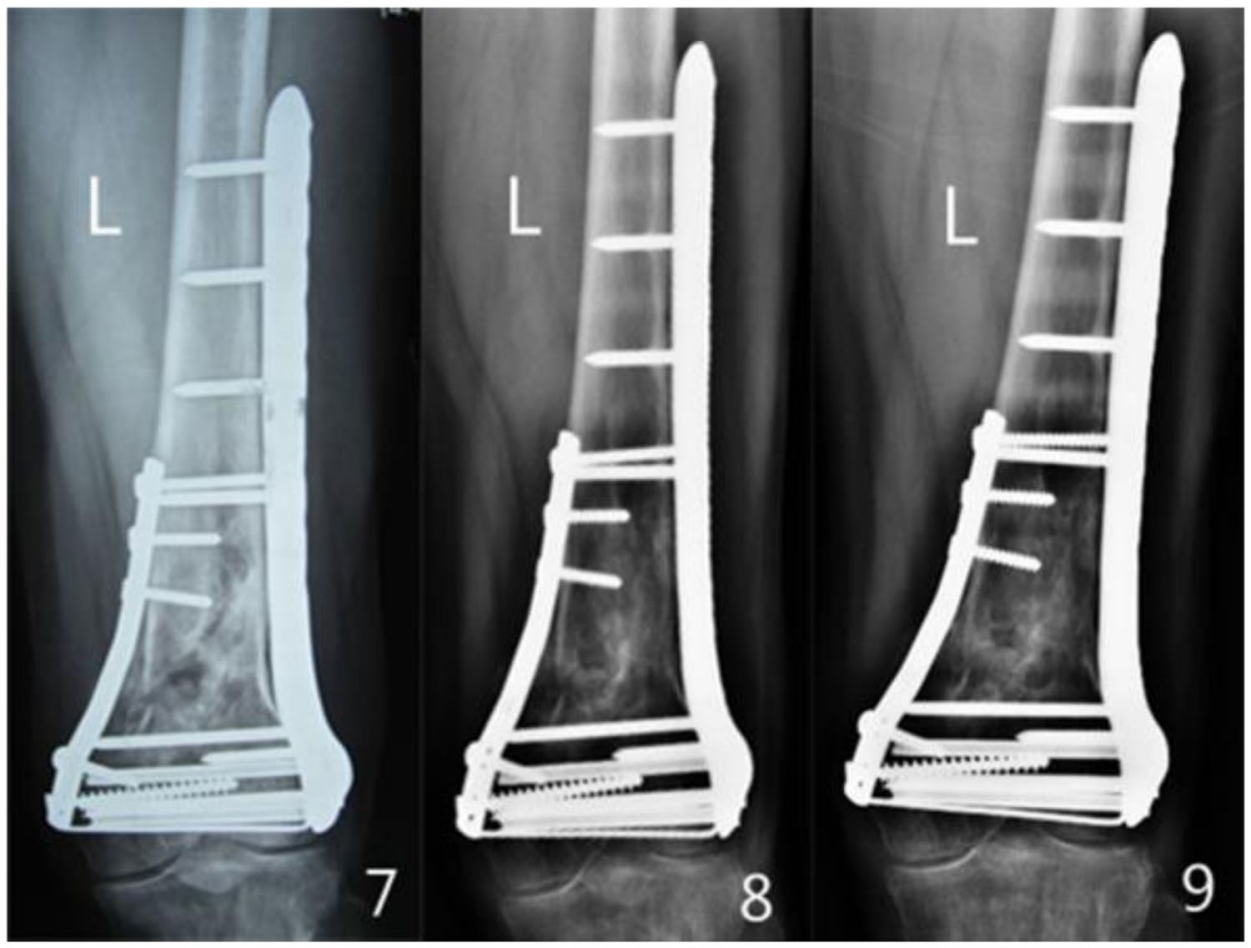
7, 8, 9 the X-ray of femur, taken by 1, 2, 3 years after operation.

**Figure 4:**
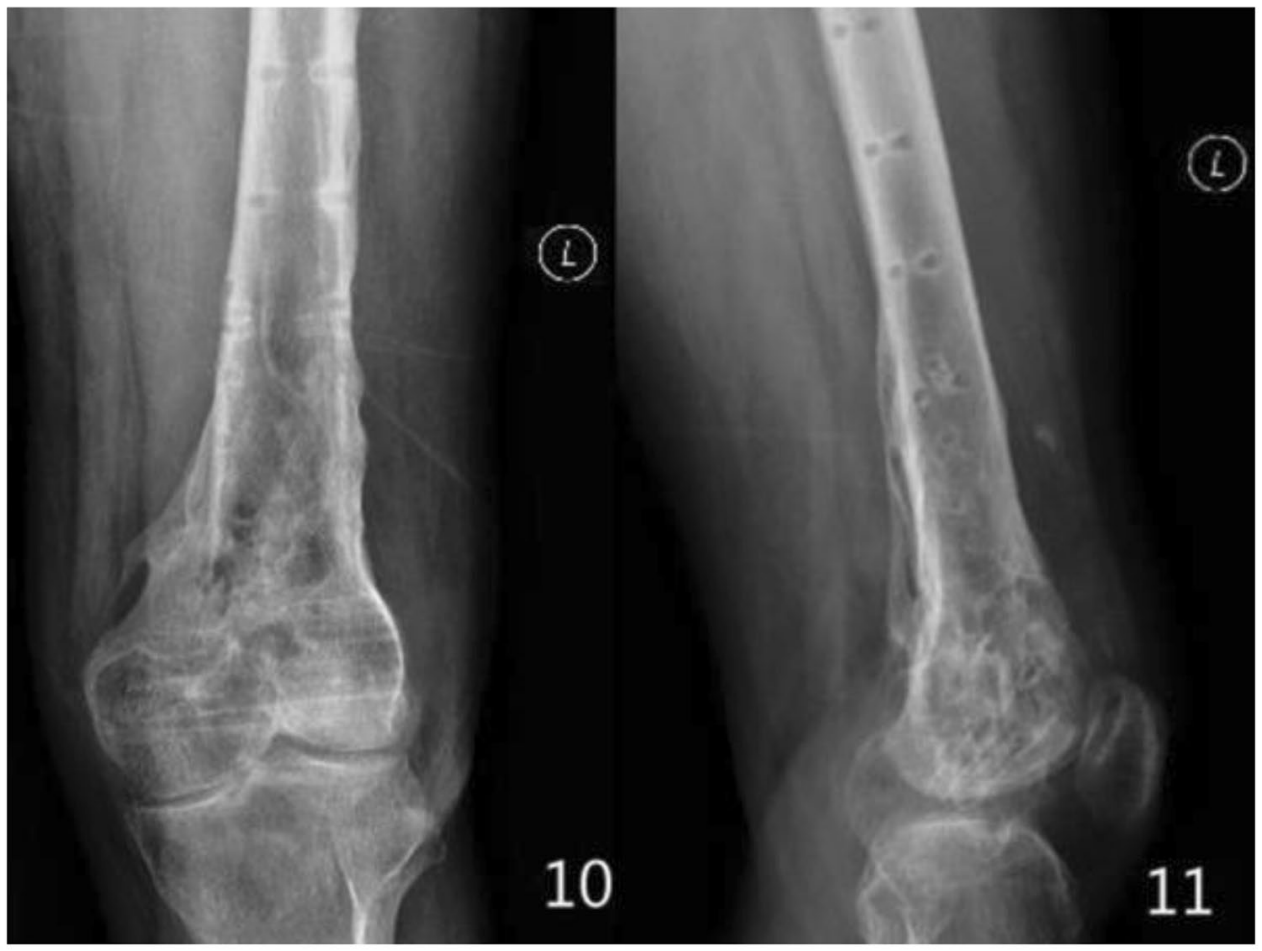
10, 11 the X-ray of the femur, taken after the plates were taken out.

**Figure 5:**
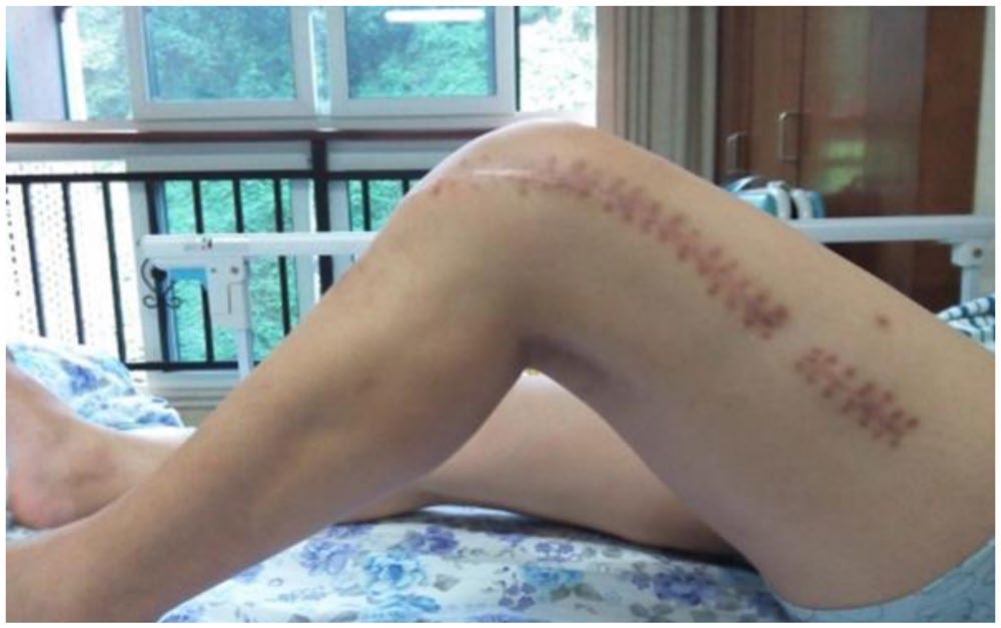
12 the knee function of the patient, taken 1year after operation.

**Figure 6:**
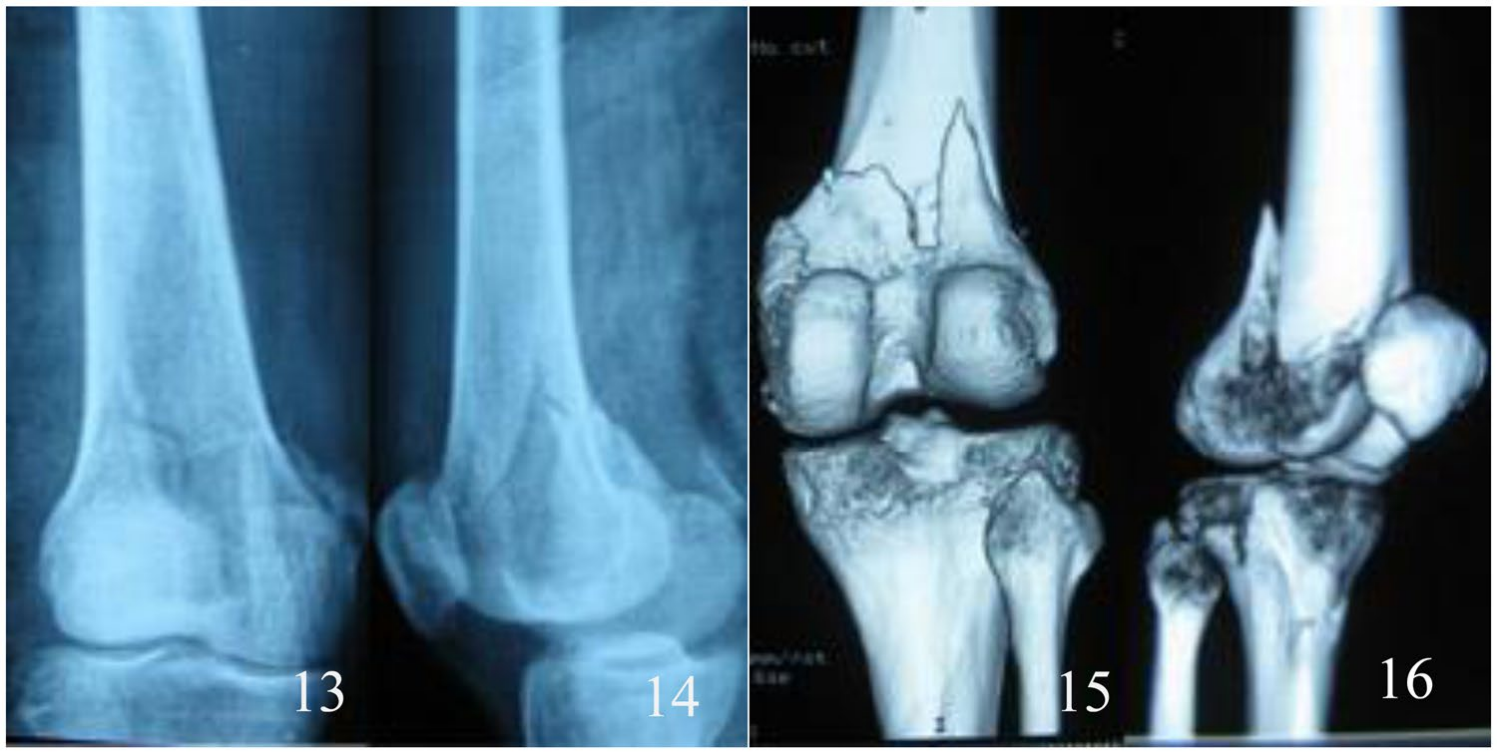
13, 14, 15, 16 the preoperative X-ray and CT three-dimensional reconstruction of femur.

**Figure 7:**
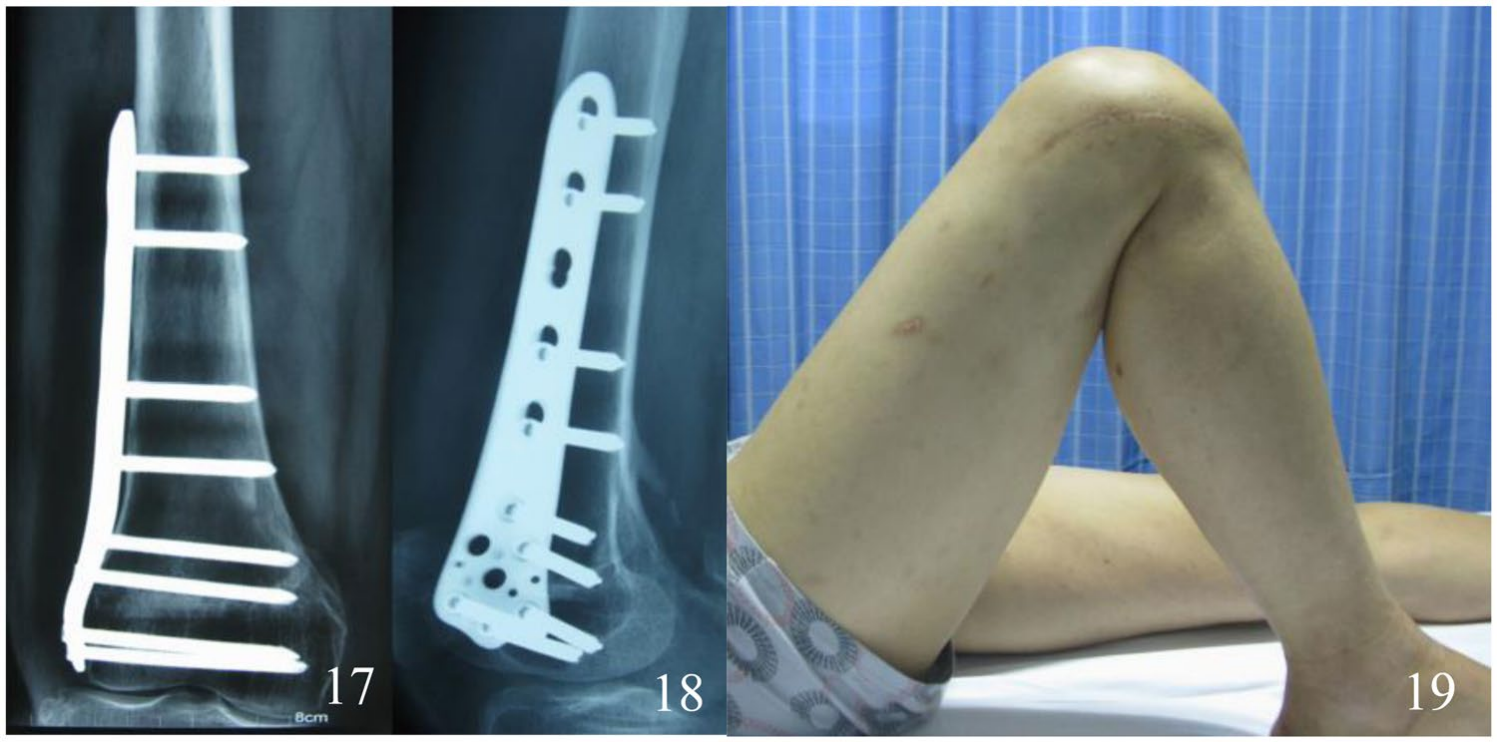
17, 18 the X-ray of femur, taken 18 months after operation, 19 the knee function of the patient, taken one year after operation.

All patients were followed up for 12 to 48 months. The follow-up records of one patient of the double-plate group are shown in Figs 1–5 and those of one patient of the single plate group are shown in Figs 6 and 7. One patient in each group died one year and a half after the operation due to other causes. There were 58 patients and 60 cases of distal femoral fractures in total, of which one patient had bilateral distal femoral fractures both fixed by lateral plate and the other one with bilateral distal femoral fractures had a single plate on the right distal femur and double plates on the left. The age distribution of patients in the single-plate group and the double-plate group is shown in Table 1. There was no significant difference between the two groups (*p* = 0.330).

**Table 1:** Age distribution of patients from single-plate and double-plate group.

age (years)	single-plate (percentage)	Double-plate (percentage)	*P*
≤30	8 (16.7%)	1 (8.3%)	0.671
31–40	8 (16.7%)	4 (33.3%)	0.234
41–50	10 (20.8%)	4 (33.3%)	0.448
≥51	22 (45.8%)	3 (25.0%)	0.327

The male and female ratio of the single-plate group was 23:25 and that of the double-plate group was 1:1. (Table 2) There was no significant difference in the male and female sex ratio between the two groups (*p* = 0.897). In the double-plate group, the percentage of cases injured in traffic accidents is 83.3%, which was higher than that of the single-plate group. While the percentage of falling injuries (16.7%) was lower than that of the single-plate group. There were significant differences in injury factors (*p* = 0.042) (Table 3).

**Table 2:** Gender distribution of patients from single-plate and double-plate group.

Gender	single-plate (percentage)	double-plate (percentage)
male	23 (47.9%)	6 (50.0%)
female	25 (52.1%)	6 (50.0%)

**Table 3:** Injury factors of patients from single-plate and double-plate group.

Injury Factors	single-plate (percentage)	double-plate (percentage)
traffic accidents	20 (41.7%)	10 (83.3%)
falling injuries	22 (45.8%)	2 (16.7%)
heavy pound	6 (12.5%)	0

There were 23 cases accounting for 48.0% of closed fractures in single-plate group. There were 9, 11 and 5 cases of type I, type II and type III by Gustilo classification in open fractures, accounting for 18.8%, 22.9% and 10.4%, respectively (Table 4).

**Table 4:** The open degree and combined injury of patients from single-plate and double-plate group.

Types	single-plate (percentage)	double-plate (percentage)	*P*
closed	23 (48.0%)	1 (8.3%)	0.019
gustilo type I	9 (18.8%)	5 (41.7%)	0.130
gustilo type II	11 (22.9%)	5 (41.7%)	0.273
gustilo type III	5 (10.4%)	1 (8.3%)	1
combined Vascular injury	2	0	—
combined external fixator	7	1	—

According to the AO classification, of the 48 cases of the single-plate group, there were 16 cases belonging to the type A, accounting for 33.3%; there were 6 cases belonging to the type B, accounting for 12.5%; there were 26 cases belonging to the type C, accounting for 54.2%, which included 2 cases of type C1, 4 cases of type C2 and 20 cases of type C3. Of the  12 cases of the double-plate group, they were all cases belonging to type C which was significantly more than that of the single-plate group (*p* = 0.009) and included 1 case of type C1, 2 cases of type C2 and 9 cases of type C3 (Table 5).

**Table 5:** The fracture types of patients, from single-plate and double-plate group.

OTA/AO	single-plate (percentage)	double-plate (percentage)
type A	16 (33.3%)	0
type B	6 (12.5%)	0
type C	26 (54.2%)	12 (100.0%)

Since some patients may also have fractures in other areas, we also classified the cases according to the locations of fractures. In single-plate group, there were 10 cases combined with 1 extra location of fractures and 13 cases combined with 2 or more extra locations of fractures, which included 6 cases caused by traffic accident, 6 cases caused by falling injury and 1 case caused by heavy pound. In the double-plate group, there were 2 cases combined with 1 extra location of fractures and no case combined with 2 or more (Table 6), which had no significant difference with the single-plate group (*p* = 0.058).

**Table 6:** The extra locations of fractures in patients from single-plate and double-plate groups.

Number of locations	single-plate (percentage)	double-plate (percentage)
1	10 (20.8%)	2 (16.7%)
≥2	13 (27.1%)	0

In the single-plate group and the double-plate group, the average follow-up time was 15.2 months and 18.5 months; the mean fracture healing time was 14.3 months and 18 months, respectively, with no significant difference (*p* = 0.559); the average time between injury and internal fixation was 11 days and 12 days; the average length of stay was both 32 days. There was no significant difference in intra-operative hemorrhage, operation time and healing time between the two groups (Tables 7, 8). In the single-plate group, the rate of bone grafting during operation was 40.4% and that of the double-plate group was 91.7% (Table 9), which was significantly different with that of the single-plate group (*p* = 0.002).

**Table 7:** The operation time, intraoperative hemorrhage, healing time of patients from single-plate and double-plate groups.

Items	single-plate	double-plate	*P*
operation time (min)	145	180	0.170
intraoperative hemorrhage (ml)	513	814	0.270
healing time (m)	14.3	18	0.559

**Table 8:** The healing rates of patients from single-plate and double-plate group.

healing condition	single-plate (percentage)	double-plate (percentage)
nonunion	1 (2.1%)	0 (0.0%)
bony union	47 (97.9%)	12 (100.0%)

**Table 9:** The bone grafting of patients from single-plate and double-plate group.

Kinds of bone grafting	single-plate (percentage)	double-plate (percentage)
no bone grafting	29 (60.4%)	1 (8.3%)
autogenous iliac bone	2 (4.2%)	7 (58.3%)
artificial bone	15 (31.3%)	1 (8.3%)
combined bone grafting	2 (4.2%)	3 (25.0%)

One case of failure in internal fixation was observed in patient in the single-plate group, which was retreated with autologous iliac bone graft and lateral anatomical plate fixation. During the follow-up, no recurrence of internal fixation failure was observed. In the double-plate group, no internal fixation loosening or rupture, no loss of fracture reduction and no knee varus deformity was observed. In the single-plate group, 1 patient was transferred to our hospital combined with the injury of the femoral artery, who received the treatment of femoral artery repair and fasciotomy in emergency. Postoperatively, the patient developed ischemic contracture and talipes equinovarus, which was corrected by second operation finally. All fractures of two groups healed clinically and no loss of reduction was observed. According to the Kolmert’s standard, there was no significant difference in excellent and good rates between the two groups (Table 10, *p* = 0.692). Of the 3 patients with poor scores by Kolmert’s standard, none was combined with ipsilateral tibial plateau fractures but 1 case was combined with periprosthetic femoral fractures and 2 cases were combined with a complete rupture of the quadriceps. One year postoperatively, the knees of the 3 patients with poor scores could be flexed about 30–50 degrees and no shortening or varus deformity was observed.

**Table 10:** The knee function of patients from single-plate and double-plate group.

Kolmert’s standard	single-plate (percentage)	double-plate (percentage)
excellent	15 (31.3%)	4 (33.3%)
good	24 (50.0%)	5 (41.7%)
fair	7 (14.6%)	2 (16.7%)
poor	2 (4.2%)	1 (8.3%)

## Discussion

In clinical, according to the OTA/AO classification, the distal femoral fractures are divided into type A: extra-articular fracture; type B: partial intra-articular fractures; type C: complete intra-articular fractures. The current treatments for distal femoral fractures are non-operative treatment, external stent fixation, intramedullary nail fixation and internal fixation with plate(s) and screws. The sixty cases with distal femoral fractures which were followed up were all treated with internal fixation with plate(s) and screws.

### Onset characteristics of distal femoral fractures

The age distribution of patients with distal femoral fractures in our study revealed that patients under 40 and over 50 are more susceptible, who accounted for 79.2% and 66.7% of the patients, respectively, which was consistent with that reported by Martinet 0^2^ and there was no significant difference between the two groups (*p* = 0.439). There was also no significant difference in sex ratio (male to female) in both groups (*p* = 0.897), which was consistent with that reported by Wu Huimin^6^. In the single-plate group, traffic accident injuries (41.7%) and falling injuries (45.8%) were the main causes, while in the double plates group patients were mainly injured by traffic accidents (83.3%), which was significantly different (*p* = 0.042). In addition, the patients who developed 2 or more extra locations of fractures accounted for 21.7% of the single-plate group but none was observed in the double-plate group. The patients with distal femoral fractures of type C (especially type C3) of the double-plate group were significantly more than those of the single-plate group (*p* = 0.009). The differences in numbers of fracture types (especially C) between two groups indicated patients in the double-plate group suffered more powerful violence during the sudden injury and that of patients who developed 2 or more extra locations of fractures between two groups revealed that the violence was possibly shared by multi locations, leading to less patient with distal femoral fractures of type C.

Open distal femoral fractures were mostly caused by car accidents (accounting for 75%) and the proportion of that in the double-plate group was 91.7%, significantly higher than 52% of the single-plate group (*p* = 0.035). Patients with open fractures with or without vascular injury were all treated in emergency. Of the two groups, 8 cases were treated with external fixation in emergency which were replaced by plate(s) and screws, one week later, of which 2 cases of vascular repair were performed intraoperatively. The occurrence of distal femoral fractures combined with vascular injury was low, accounting for 3.3%, similar to the 3% reported by Fu^7^.

With the increasing number of patients developing periprosthetic femur fractures after receiving knee arthroplasty, it is still unclear whether the fracture around prosthesis is directly related to knee arthroplasty but it was reported that the prognosis of patients with periprosthetic femur fracture treated with plate(s) and screws was better than those treated with intramedullary nail^8–16^.

### Comparison of clinical efficiency of single plate and double-plating fixation of distal femoral fractures

In the double-plate group, average amount of intraoperative hemorrhage, average operation time, and average fracture healing time were similar to that of the single-plate group, while the occurrence of bone grafting was significantly higher than that of the single-plate group (*p* = 0.002). The higher occurrence of bone defect of the double plate group is consistent with the higher occurrence of distal femoral fractures of type C. Double-plating fixation leads to more damage to blood supply but does not significantly prolong the time required for fracture healing.

Of the 60 cases followed up, the wound all healed well. Of the 3 patients who developed poor knee function after the operation, the patient with periprosthetic femur fracture had a stiff knee before the operation and the other two developed femoral quadriceps tendon rupture during the injury, but none developed tibial plateau fractures. Knee functions of the 4 patients with distal femoral fractures combined with tibial plateau fractures were evaluated by the Kolmert rating system, which showed that 3 were graded as good and 1 was graded as fair. Therefore, we think postoperative knee flexion dysfunction probably be related to the femoral quadriceps tendon rupture insteadind of the tibial plateau fractures. That’s because femoral quadriceps tendon will adhere to the anterior surface of the distal femur and suprapatellar bursa during the process of healing, seriously affecting the knee function of flexion, which was conformed with what we saw during the removal surgery for internal fixation. During the surgery, when we removed the distal femoral plate(s) and screws and released the knee joint adhesions, it was often found that the adhesions were mainly outside of the joint. Although the femoral quadriceps tendon was loosened during operation, the efficacy of the adhesion release would still be reduced by the scar tissue. The rate of failure of internal fixation of distal femoral fractures was differently reported by different scholars. According to the report of AAOS in 2012, the rate of failure was 19–22%^17^. However, the occurrence of nonunion after internal fixation of distal femoral fractures reported varies a lot, of which the highest is up to 40%^18–22^. The occurrence of postoperative nounion we observed in this follow-up was low, accounting for 1.7%. The possible reasons are: Firstly, after finishing the set of lateral locking plate, we routinely performed the drawer and the lateral stress test and the medial plate was added if the varus stress test was positive; Secondly, one stage bone grafting benefits fracture healing. After comparing the clinical efficiency of single plate internal fixation with lateral and medial double-plating fixation of distal femoral fractures of type C3, Wu *et al*.^6^ found that knee function of the patients treated with lateral and medial double-plating fixation were significantly better. In our study, the result of lateral stress tests performed intraoperatively was taken as the indication of setting the lateral and medial plates insteading of the fracture type and no significant difference in excellent and good rates of postoperative knee function between the two groups was observed (*p* = 0.692).

### Some experience of minimally invasive treatment of distal femoral fractures by locking plate(s)

Treatment of distal femoral fractures with lateral locking plate causes less damage to the soft tissue and blood supply of the medial side of distal femur, reducing the risk of intraoperative injury of the femoral artery and femoral nerve. With a wider indication than treatment with intramedullary nail, treatment with locking plate can also be used in intra-articular comminuted fractures^23^. What’s more, it also causes less loss of reduction than treatment with dynamic compression plate^24^ and enables the patient to exercise the knee function earlier than treatment with external fixation^25,26^. However, when fracture fragments in the medial side of distal femur were seriously comminuted or with a massive bone defect, the single lateral plate fixation of the distal femoral fractures may fail to stabilize fracture sites, leading to knee varus deformity, looseness and breaking of plate and screws and nonunion. In terms of lateral and medial double-plating fixation of distal femoral fractures, lateral and medial incisions which are made for setting the plates makes it easier for reduction and correcting the line of force, leading to a low occurrence of knee varus deformity and loss of reduction^27^. Jiang *et al*.^28^ followed up 21 patients with distal femoral fractures treated with lateral and medial double-plating fixation, no case of knee varus deformity postoperatively was observed, which was consistent with our study. The implant of the medial plate may damage the blood supply of the medial side of fracture sites, which may increase the fracture healing time, intraoperative hemorrhage and operation time. In this follow-up, although the average time of fracture healing in double-plate group was longer than that of the single-plate group, it was not statistically different.

The indication of using double-plating fixation of distal femoral fractures was inspired by the treatment of patients who developed loosening and rupture of plate and screws or knee varus deformity after operation. During the operation, the varus stress tests performed were all positive and they turned into negative after setting the medial plate. After operation, fractures healed without knee varus deformity. Bottlang *et al*.^4^ once fixed the fracture model with bone defect of about 1 cm by anatomical locking plate, then applied the axial force of 400 N, the displacement of the cortical was found no more than 0.3 mm, included in the allowed range of micro-movement (0.2–1 mm). Therefore, intraoperative bone graft is suggested for bone defect of more than 1 cm.

In summary, based on our research, we suggest that after the placement of the lateral locking plate for fixing distal femoral fractures, if the knee joint varus stress test is positive and rupture of the lateral collateral ligament is excluded, the medial plate should be used to increase the stability of fractures to avoid postoperative fracture nonunion and knee varus deformity. Besides, lateral and medial double-plating fixation doesn’t significantly increase or influence the operation time, intraoperative hemorrhage, fracture healing time and postoperative knee function.

### Population

Inclusion criteria: Patients with distal femoral fractures treated with locking plate(s). Exclusion criteria: Patient with a decline of muscle strength of lower extremities. Anterior-Posterior and lateral plain film, computed tomography and 3D reconstruction of the fracture sites were performed to identify the fracture types.

There were 48 cases of fractures treated with lateral plate (single-plate group): female in 25 cases, male in 23 cases, traffic accidents in 20 cases, falling injuries in 22 cases and heavy pound in 6 cases; according to OTA/AO classification: type A in 16 cases, type B in 6 cases and type C in 26 cases (type C1 in 2cases, typeC2 in 4 cases and type C3 in 20cases).There were 12 cases of fractures treated with lateral and medial double-plating fixation (double-plate group): female in 6 cases and male in 6 cases; traffic accidents in 10 cases, falling injuries in 2 cases; according to OTA/AO classification: type C1 in one case, type C2 in 2 cases and type C3 in 9 cases. Informed consents for both study participation and publication of identifying information/images in an online open-access publication were obtained from the participants. The study was approved by the ethic committee of the first affiliated hospital of Chongqing medical university. All procedures performed in studies involving human participants were in accordance with the ethical standards of the institutional and/or national research committee and with the 1964 Helsinki declaration and its later amendments or comparable ethical standards.

### Material

The lateral locking plate was produced by Zimmer or Synthes company and the medial plate used was an anatomical plate on the medial side of distal femur or upper limber compressing plate.

### Preoperative preparation

Patients with open fractures were all treated with debridement and suturing in emergencies, of which the cases with fractures combined with vascular injury were all treated with vascular repair and external fixation during operation. All external fixations were changed to internal fixation one week later. All closed distal femoral fractures were treated with skeletal traction and cold therapy with lesion legs lifted and fixation was performed when the swelling subsided.

### Operative method

All operations were performed by the same group of surgeons and patients of both groups were under general anesthesia.

Single-plate group: The anterior lateral incision of femur was made and the fracture sites were exposed. The reduction of medial, lateral condyles, intercondylar and supracondylar fractures were performed one by one and Kirschner wires were used for temporary fixation. One stage bone grafting was performed for bone defects more than 1 cm. The lock plate with aiming sight was set under the muscle along the surface of the femur. After the temporary fixation on the distal end with Kirschner wire, guide pin was drilled into the proximal end of the fracture sites to keep the positions of fractures and plate. Assisted by x-ray examination of the fracture sites during operation, the screws were implanted correctly and kept away from the articular cavity. Then stability of the knee was evaluated by anterior and posterior draft test and stress test. If the varus stress test turned out positive and rupture of the lateral collateral ligament was excluded, the medial plate was set. Finally, we washed the wound, placed a negative pressure drainage tube, closed the incision layer by layer, and bandaged the leg up with an elastic bandage.

Double plates group: The lateral plate was set as the single-plate group. When setting the medial plate, the arcuate incision in the medial side of leg was made. Separated by layer by layer, the inferior segment of the femur was revealed while the vastus medialis were retained. Then the medial plate was implanted, fixed by no less than two screws on the distal fracture site. Finally, the knee varus stress test turned into negative.

### Postoperative management

Prevention of infection, analgesia and anticoagulation for 7–10 days were routinely performed. The drainage tube was usually pulled out within 72 hours after operation. For patients with rigid internal fixations, isometric contraction exercises of the quadriceps and continuous passive motion (CPM) of the knee were suggested immediately after surgery. Active knee flexion and extension exercises were suggested, 72 hours after operation. For patients with only distal femoral fractures, non-weight bearing walking with the aid of double axillary crutches was suggested 5–7 days after operation.

### Outpatient follow-up

Plain film was performed every 3 months after operation for monitoring the fracture healing and whether there was loss of reduction and rupture of plate(s) and screws. Each patient was followed up until the fracture union and no less than one year. According to the plain film and condition of the patient, it was determined when partially loaded walking should be started and how to exercise step by step. Knee function was evaluated by Kolmert’ standard, one year after surgery.

### Statistical analysis

Statistical analysis was performed with IBM SPSS Statistics 21.0 software. The measurement data were analyzed by rank sum test and the count data were analyzed by the chi-square test. A P-value < 0.05 was significant.
